# Alternative observational designs to estimate the effectiveness of one dose of oral cholera vaccine in Lusaka, Zambia

**DOI:** 10.1017/S095026882000062X

**Published:** 2020-03-13

**Authors:** E. Ferreras, A. Blake, O. Chewe, J. Mwaba, G. Zulu, M. Poncin, A. Rakesh, A. L. Page, M. L. Quilici, A. S. Azman, S. Cohuet, I. Ciglenecki, K. Malama, E. Chizema-Kawesha, F. J. Luquero

**Affiliations:** 1 Epicentre, Paris, France; 2 Ministry of Health, Lusaka, Zambia; 3 Zambia National Public Health Institute, Lusaka, Zambia; 4 Department of Pathology and Microbiology, University Teaching Hospital, Lusaka, Zambia; 5 Centre for Infectious Diseases Research in Zambia, Lusaka, Zambia; 6 Médecins Sans Frontières, Geneva, Switzerland; 7 Institut Pasteur, Paris, France; 8 Department of Epidemiology, Johns Hopkins Bloomberg School of Public Health, Baltimore, MD, USA; 9 Department of International Health, Johns Hopkins Bloomberg School of Public Health, Baltimore, MD, USA

**Keywords:** Cholera, infectious disease epidemiology, public health, vaccine policy development

## Abstract

We conducted a matched case-control (MCC), test-negative case-control (TNCC) and case-cohort study in 2016 in Lusaka, Zambia, following a mass vaccination campaign. Confirmed cholera cases served as cases in all three study designs. In the TNCC, control-subjects were cases with negative cholera culture and polymerase chain reaction results. Matched controls by age and sex were selected among neighbours of the confirmed cases in the MCC study. For the case-cohort study, we recruited a cohort of randomly selected individuals living in areas considered at-risk of cholera. We recruited 211 suspected cases (66 confirmed cholera cases and 145 non-cholera diarrhoea cases), 1055 matched controls and a cohort of 921. Adjusted vaccine effectiveness of one dose of oral cholera vaccine (OCV) was 88.9% (95% confidence interval (CI) 42.7–97.8) in the MCC study, 80.2% (95% CI: 16.9–95.3) in the TNCC design and 89.4% (95% CI: 64.6–96.9) in the case-cohort study. Three study designs confirmed the short-term effectiveness of single dose OCV. Major healthcare-seeking behaviour bias did not appear to affect our estimates. Most of the protection among vaccinated individuals could be attributed to the direct effect of the vaccine.

## Introduction

Observational studies are often used to better understand how vaccines perform across a variety of real populations with different epidemiologic and geographical settings; especially, as new vaccines are introduced into routine immunisation schedules and the number and timing of dose changes. The evidence base produced from these evaluations is key to setting vaccine policy [1, 2]. Evaluation of vaccine effectiveness (VE) typically requires detecting and recruiting individuals with the disease of interest. For many epidemic-prone diseases, such as cholera, outbreaks provide a critical window where vaccine evaluations may be feasible [3–5].

Among the study designs to evaluate VE during outbreaks [6], case-control studies are the most used because of their practical design for rare diseases, as the odds ratio (OR) approximates the relative risk (RR) and therefore can be used to estimate VE [7, 8]. These studies are relatively quick to conduct and less expensive than cohort studies. In the traditional case-control design, controls are randomly selected members of the study population who have not developed the disease of interest prior to their inclusion. In the test-negative case-control (TNCC), analysis is limited to those seeking healthcare for similar symptoms; with controls being those who test negative for the disease of interest. Test-negative controls are a good alternative in emergency situations because of their time efficiency [9]. The case-cohort is a variant of the case-control design where controls are randomly sampled from the initial population at-risk, and may thus include both cases and non-cases. Unlike the case-control design, sampling is done *a priori* without regards to case status or time, providing an estimate of the RR [10]. Moreover, this approach allows taking into account the variable person-time at-risk in vaccinated and unvaccinated states and provides a VE estimate incorporating some degree of indirect effects [3].

Irrespective of the study design selected, an accurate estimate of VE requires the accurate ascertainment of susceptibility to the infection, vaccination status and disease status among the study population and comparability in other characteristics among vaccinees and non-vaccinees [2]. The absence of randomisation between study groups in observational studies may lead to differences that affect their risk of infection, for reasons other than their vaccination status. If not adequately measured and adjusted for in analyses, these differences may confound the association between vaccination and the outcome.

Confounders are associated with both probability of vaccination and with the outcome. Solutions at the design level, such as matching, or at the analysis level, through stratification or multi-variable analysis, can control confounding. In matched case-control (MCC), community control recruitment facilitates matching on key confounders such as age, neighbourhood and time [11]. Spatial matching helps controlling for local variations not only in risk but also in vaccination coverage.

In MCCs, where controls are selected from the community, it is possible that they have different access to care or healthcare-seeking behaviour compared to cases, which could lead to biased estimates of VE. This is not the case for the TNCC controls, since they are selected following care seeking [12]. However, TNCC is more sensitive to disease misclassification and, therefore, risk of selection bias [13].

In February 2016 in Lusaka, Zambia a cholera outbreak was declared after 4 years with no confirmed cholera in the city. A reactive oral cholera vaccine (OCV) campaign [14] was carried out between 9 and 25 April 2016 [15]. Due to a global vaccine shortage, the Ministry of Health with support from Médecins Sans Frontières (MSF) and the World Health Organization decided to use a single dose regimen of OCVs to vaccinate high-risk areas to halt transmission within Lusaka and limit the probability of spread within the country.

**Fig. 1.**
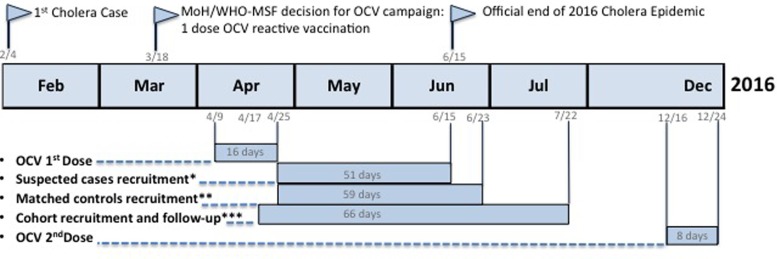
Timeline, Lusaka, Zambia, 2016. *Test-negative; **case-control; ***case-cohort.

We conducted a VE study in Lusaka, Zambia (Fig. 1), using three methodologies: MCC, TNCC and case-cohort design, to quantify the short-term protection provided by one dose of OCV (Shanchol^®^, Shantha Biotechnics, Hyderabad, India). This setting provided a unique opportunity to measure the effect of the vaccine comparing estimates of VE from different designs to better understand the robustness of conclusions from the primary analysis published elsewhere [4] and to inform the design of future observational studies of OCV VE accounting for different possible sources of biases.

## Methods

### Study area and cholera surveillance

Cholera is endemic in Zambia. The high risk areas in Lusaka, defined from historical data and access to water and sanitation conditions, represent around 35% of the city's population, mainly in the western peri-urban areas of the city [16]. From 14 April 2016 until the end of the outbreak, the surveillance system was reinforced with the use of standardised line-lists and case definitions in the five cholera treatment centres (CTC), located in the higher risk areas for cholera.

### Definitions and laboratory confirmation

A suspected cholera case was any person admitted in any of the CTC between 25 April and 15 June 2016 (official declaration of the end of the outbreak) with acute watery diarrhoea (at least three watery stools in a 24-h period). All suspected cholera cases were included in the study if (1) resided in the study area since 9 April 2016 (first day of vaccination (FDV)); (2) was older than 12 months on FDV; (3) diarrhoea started after FDV and (4) her/his residence could be located by the study team after discharge.

During admission, a stool sample was collected from all suspected cases in an unused and unchlorinated container. Fresh stools were used to perform culture on site, at Kanyama clinic laboratory, using standard methods [17]. If written consent was obtained, two drops of stool sample were placed on Whatman 903 filter paper for subsequent polymerase chain reaction (PCR) testing at the University Teaching Hospital (Lusaka, Zambia) and Institut Pasteur (Paris, France), using previously described methods [18].

All suspected cholera cases were included and divided into two groups based on cholera culture/PCR results. Confirmed cholera cases (culture and/or PCR positive result) were compared with non-cholera diarrhoea controls (negative to culture and PCR).

A neighbour of the same sex and within the same age group (1–4, 5–9, 10–19, 20–29, 30–39 and ≥40 years) as the confirmed cholera case was eligible to be a matched control if s/he: resided in the study area since FDV; was at least 12 months; had not sought treatment for diarrhoea between 1 January 2016 and the date of onset of the matched case's diarrhoea and would have sought treatment in a CTC if severe, watery diarrhoea had developed.

### Study design

We performed a case control study and a case-cohort study. After obtaining signed consent, study staff conducted a structured face-to-face interview with each suspected case at the CTC between 25 April and 15 June 2016. After interviewing the cases, five neighbour-controls were selected per confirmed cholera case and were interviewed at their houses the same week their matched case was interviewed. In cases where the participant was a minor, study teams interviewed the parents/guardian in the presence of the minor, when possible.

From 17 April to 25 May 2016 we recruited a cohort of 906 randomly selected individuals living in areas considered at high risk of cholera transmission (some of them targeted and some of them non-targeted by the reactive OCV campaign). Cohort members were selected from townships with a probability proportional to their population size. We randomly selected households by drawing GPS positions in georeferenced polygons of the township boundaries [19]. One person aged 12 months and above, from each household was randomly chosen. Participants had same eligibility criteria than described above.

Study staff first interviewed the selected cohort participants at their houses and then followed them up to 24 July 2016. The occurrence of episodes of diarrhoea, both medically attended and non-medically attended, and vaccination of non-previously vaccinated participants was checked during the follow-up. Study participants recorded on the master national line list of suspected cholera cases at the end of the study would be considered as cases.

Participants were asked whether they had been vaccinated – including when, where and whether it was completely ingested – after showing a picture of a vaccine vial and of an adult taking the vaccine. The interviewers provided to each participant details of the vaccination campaign to ensure an adequate identification of the antigen. The vaccination card was systematically checked and photographed if it was provided.

Vaccination status, clinical, demographic, socioeconomic and environmental variables were ascertained through electronic questionnaires using Kobo Toolbox software 1.4.8 (Cambridge, MA, USA).

### Analysis

The primary analysis of VE was based on the MCC design, which has been published elsewhere [4]. Here, we reanalysed the data adding two more approaches to estimate the effectiveness of one dose of OCV: a TNCC and a case-cohort design.

We assessed the protection conferred by the intake of one dose of vaccine against stool culture or/and PCR confirmed cholera. In our analyses a person was considered to be vaccinated 7 days after ingesting the vaccine (without spit/vomit).

For the TNCC and the MCC, we compared the odds of vaccination between confirmed cholera cases and controls (non-cholera diarrhoea or matched) using univariate and multivariable conditional logistic regression models. We calculated the VE as (1 − OR) × 100.

In the multivariate analysis, we explored the potential confounding effect of different well-known risk factors for cholera (Supplementary Tables S1 and S3) to obtain adjusted VE estimates (Table 1). We defined variables as possible confounders when they were associated (*P*-value <0.2) with the outcome and with the exposure (vaccination status). We also considered as potential confounder variables that modified the VE in more than 5% in the bivariate models (including vaccination status). We included all possible confounders in the final adjusted model.

**Table 1.** Crude and adjusted VE estimates

	Vaccinated (single-dose)^a^	Unvaccinated	Crude VE		Adjusted VE	
	No. of participants (%)		(95% CI)	*P* value	(95% CI)	*P* value
TNCC analysis						
Cholera cases	3 (5)	63(95)	Ref		Ref	
Non-cholera diarrhoea cases	39 (27)	106 (73)	87.1% (62.4–97.0)	<0.01	80.2%^b^ (16.9–95.3)	0.03
MCC analysis						
Cholera cases	3 (5)	63 (95)	Ref		Ref	
Matched controls	44 (13)	286 (87)	84.7% (27.0–96.6)	0.02	88.9%^c^ (42.7–97.8)	<0.01
Case-cohort analysis						
Cholera cases	3 (5)	63 (95)	Ref		Ref	
Person time at-risk (in days)	48 765.6	18 826.0	86.7% (56.6–95.9)	<0.01	89.4%^a^ (64.6–96.9)	<0.01

a Case-cohort: VE was adjusted by age, sex, number of children under 5 years of age living in the household, access to safe water and the place of defecation.

b TNCC: VE was adjusted by age, education level, frequency of treating the drinking water and contact (combined variable that considers those who had a household member with cholera in the previous week or shared the drinking-water source with a cholera patient as ‘exposed’). Living in a vaccinated area was included as a stratification variable in the conditional logistic regression model.

c MCC: Adjusted by contact. Living in a vaccinated area was included as a stratification variable in the regression model.

All *P*-values and 95% confidence interval (CI) were two-sided. We used R statistical software (version 3.2.3) and the *survival* package for the main analyses. Missing data were treated as described in the Supplementary material.

For the case-cohort, we estimated unadjusted and adjusted hazard ratios (HR) of medically attended cholera comparing those who received the vaccine to those who did not. The HR was then translated into overall VE by using the formula: VE = (1 − HR) × 100. We used proportional hazard models with vaccination as an independent variable and a time origin of 9 April 2016 (FDV). Cases that did not come from the cohort contributed person-time 0.01 days before his/her time of symptom onset following standard case-cohort analyses [10]. To be conservative, individuals in the cohort reporting diarrhoea during study follow-up with no evidence of confirmed cholera (through matching with the line list) remained ‘at-risk’ for cholera after the date of diarrhoea. We explored violations of non-proportionality of hazards visually and through generalised regression of the Schoenfeld residuals of vaccination with (log) time [20].

Adjusted estimates used multiple imputation of missing values for all designs.

### Ethical aspects

The study protocol was approved by the Ethical Review Boards of the University of Zambia and the Johns Hopkins Bloomberg School of Public Health (USA). Privacy and confidentiality of the data collected from participants was ensured both during and after the study. Informed consent was signed by all participants.

## Results

**Fig. 2.**
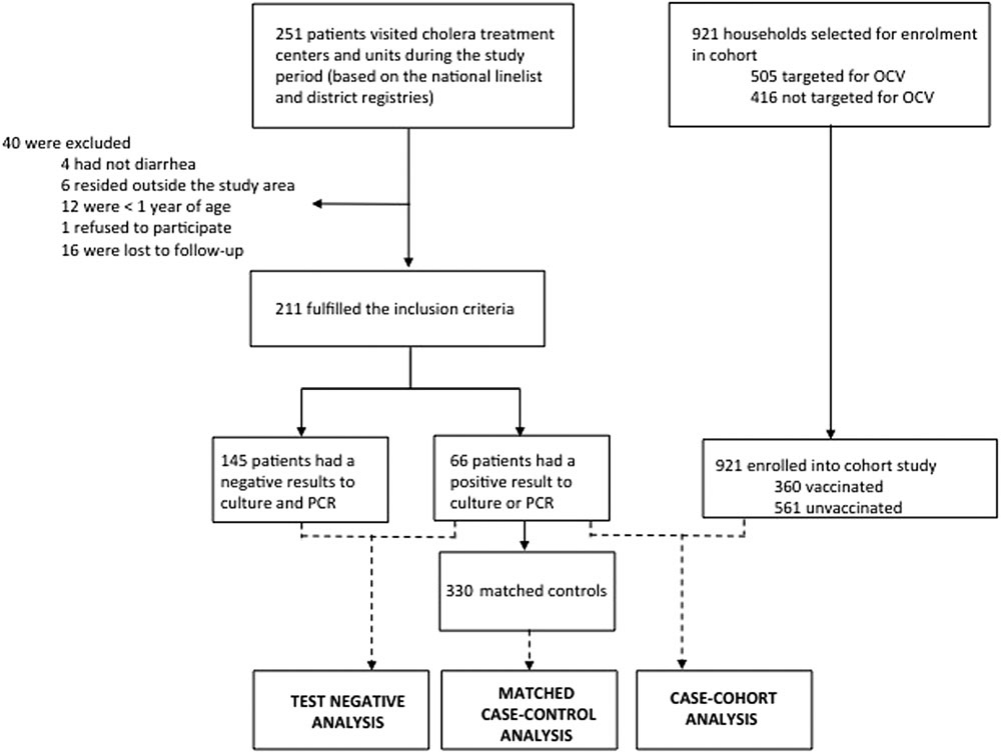
Study flowchart.

From 25 April to 15 June 2016, 251 patients with acute watery diarrhoea were admitted and treated at health centres in the study area and of these, 211 of these suspected cholera fulfilled inclusion criteria (Fig. 2).

The mean age of the suspected cholera cases included in the study was 21.6 years (s.d.: 18.2), with 28% (*n* = 59) of them being children under 5 years. Half (50%) of the suspected cases were females, although among confirmed cases, 42% were females. A total of 82% of the cases had some degree of dehydration at admission (55% were severely dehydrated). Severe dehydration was more common among confirmed cholera cases than among non-cholera diarrhoea cases (89% *vs.* 39%) (Supplementary material).

### Test-negative case-control analysis

Among the 211 suspected cholera cases included in study, 66 (31%) had positive culture and/or PCR results for *Vibrio cholerae* O1, 63 unvaccinated and 3 vaccinated. All confirmed cases had *V. cholerae* O1 serotype Ogawa. From the 145 non-cholera diarrhoea cases, 39 were vaccinated and 106 unvaccinated. Other socio-demographic characteristics of the confirmed cholera cases and the non-cholera diarrhoea cases are detailed in Supplementary Table S1. Using unadjusted and adjusted logistic regression models, VE was 87.1% (95% CI: 62.4–97.0) and 80.2% (95% CI: 16.9–95.3), respectively (Table 1).

### Matched case-control analysis

In total, 330 matched controls were interviewed of which 44 were vaccinated. The median distance between cases and their matched control households was 49 m (interquartile range: 16.8–119). Characteristics of the confirmed cholera cases and their matched controls were similar except that cases more frequently reported household members with recent cholera and sharing a water source/latrine with cholera/diarrhoea patients (Table S1).

Vaccination with one dose of OCV was associated with protection against cholera, in the crude analysis (VE = 84.7%; 95% CI: 27.0–96.6) and after adjustment for potential confounders (VE = 88.9%; 95% CI: 42.7–97.8) (Table 1).

### Case-cohort analysis

Overall, 360 vaccinated and 561 unvaccinated individuals were recruited as part of the cohort (100% participation). A total of 811 participants (88%) were successfully contacted for follow-up at the end of the outbreak. Twenty-eight cases of acute watery diarrhoea were reported within this cohort, but none of them required admission to a health structure and none appeared in the CTC registers. Sixty-six cholera confirmed cases recruited through passive surveillance were included in the case-cohort analysis (same as in the MCC and TNCC).

The crude VE using the case-cohort design was 86.7% (95% CI: 86.6–95.9) and the adjusted VE was 89.4% (95% CI: 64.6–96.9) (Table 1).

## Discussion

This study aims to measure the short-term protection of a single dose of OCV. The three study designs found comparable levels of protection and confirm that single dose of OCV confers high protection against medically attended cholera infection for at least 2 months following immunisation.

A recent study in Odisha (India) that used a TNCC as their main analysis and a cohort analysis used to validate the main results showed that the incidence of non-cholera diarrhoea among vaccinees was 2.7 times higher than among non-vaccinees, indicating different risk of diarrhoeal diseases or heterogeneity in healthcare-seeking behaviour between vaccinees and non-vaccinees [21].

Our analysis using the TNCC design found comparable VE estimates as the MCC. It provides reassurance that major healthcare-seeking behaviour bias was not at play. Moreover, the indicator bias analysis showed that the odds of vaccination did not vary significantly between non-cholera diarrhoea cases and their controls, as described earlier [4].

Self-reported vaccination was then not associated with non-cholera diarrhoea, which supports the robustness of our VE estimates regarding healthcare-seeking behaviour bias.

We considered the analyses restricting the non-cholera diarrhoea to those with moderate to severe diarrhoea and found no differences in the estimation but in IC because of the reduced sample size. Unfortunately, we did not enrol enough cases to obtain stable VE estimates when stratifying by age and severity to explore possible effect modification.

Similarly, the VE estimates from our case-cohort study were similar to those reported in the primary MCC analyses. Spatial matching, if done at the appropriate scale, should control for the differences in vaccine coverage thus providing an estimate of the direct vaccine protection [22]. The similarity of the VE from both designs suggests that the protection among vaccinated individuals was mostly attributable to the direct effect of the vaccine in Lusaka. However, we cannot discount the possibility that the spatial matching was imperfect, allowing the inclusion of controls from areas with different vaccine coverage, and thus allowing the inclusion of indirect protection in the MCC VE estimates. A sensitive analysis with different neighbourhood sizes during a cohort study in Zanzibar suggest that herd protective effect remained stable up to the size of 500 m radius neighbourhood [23]. In our sensitive analysis, the estimates remain stable when removing those with a distance more than 150 m and same for those with a distance more than 300 m.

Our VE estimates are almost identical to the short-term single-dose effectiveness estimated from a case-cohort study in South Sudan (87%) [3] but are higher than efficacy estimated in a randomised clinical trial in Bangladesh against severe cholera (63%) [24]. Several factors might explain this difference, including different follow-up periods, different age distributions (in our study, and that of South Sudan, tended to be much older (17% under 5 years old) than in the Bangladesh study (58% of cases under 5 years old)), severity profile of cases (our study includes proportionally more severe cases) and different dominant modes of transmission. Furthermore, we cannot exclude that herd protection was confounding the true direct effect of cholera vaccine in Bangladesh, since studies have consistently shown higher direct protection in areas with lower vaccination coverage [25].

Two main biases may affect our results; misclassification and selection bias linked with the non-random distribution of the vaccine. To reduce the chances of misclassifying case status, we used both culture and PCR, which is more sensitive and robust to antibiotic use before sample collection. In addition to the 62 cases confirmed by culture, we detected four additional cases by PCR that were negative by culture. During control ascertainment, we exclude those who sought treatment for diarrhoea between 1 January 2016 and the date of onset of the matched case's diarrhoea because they were not at-risk and might bias the VE estimation. Learning from previous vaccination studies [3, 5], we set up several procedures to limit the risk of misclassification of vaccination status, including the use of visual aids and asking for proof of vaccination. Unfortunately, the vaccination card retention (52%) was lower, due to the poor quality of the vaccination cards, than previously published studies [5, 26]. It is possible that study participants were not representative of the at-risk population; however, our wide coverage of enhanced surveillance in the key areas of Lusaka, combined with the fact that vaccine coverage in the controls was similar to vaccine coverage estimated in the general (targeted) population, provides reassurance that these biases were minimal.

## Conclusions

Our three different designs provide similar estimates of high level of short-term protection for one dose of OCV and confirm that this could be an effective tool to prevent cholera during outbreaks, even in areas with little to no recent exposure to cholera and provides high short-term protection. This finding is important to support recommendations for the use of the vaccine in response to outbreaks, where high-levels of short-term protection can greatly determine the impact of any campaign [4].

Estimates from MCC design are more precise because we maximised statistical power and efficiency by matching up to five community controls to each case by age group, neighbourhood and calendar time. Alternative study designs (TNCC and case-cohort) yielded similar estimates of VE than MCC design with the advantage of having a better control related to healthcare-seeking behaviour bias and to detect eventual indirect effects of the vaccine. The TNCC are a good alternative in emergency situations, especially if community controls recruitment is compromised (i.e. security reasons). Case-cohort design is an adequate option when there are few cases after vaccination to improve the study power.

Cholera outbreaks are still a major public health threat, and nowadays OCV is considered as part of the public health measures available for outbreak response. But refining the assessment of the impact of OCV with observational study design, especially to measure the long-term protection conferred by a single dose of OCV, is still necessary to learn how to use best cholera vaccines.

## Supplementary material

For supplementary material accompanying this paper visit https://doi.org/10.1017/S095026882000062X. click here to view supplementary material
