# Supplementary material for: Alternative observational designs to estimate the effectiveness of one dose of oral cholera vaccine in Lusaka, Zambia
Source: Epidemiol Infect. 2020 Mar 13;148:e78. doi: 10.1017/S095026882000062X (PMC7163804; doi:10.1017/S095026882000062X)
Supplement: Supplementary file 1 [file S095026882000062Xsup001.docx]

*Epidemiology and Infection*

# Alternative observational designs to estimate the effectiveness of one dose of oral cholera vaccine in Lusaka, Zambia.

E. Ferreras^1*^, A. Blake^1^, O. Chewe2 ^2,3^, J. Mwaba^4, 5^, G. Zulu^2^, M. Poncin^6^, A. Rakesh^1^, A. L. Page^1^, M. L. Quilici^7^, A. S. Azman^8^, S. Cohuet^1^, I. Ciglenecki^6^, K. Malama^2^, E. Chizema-Kawesha ^2^, F. J. Luquero^1,9^

**Supplementary Material**

# Characteristics of participants

The household size was larger for case-patients with cholera confirmed by culture than among those with non-cholera diarrhea. They had more often an individual with cholera in their households the previous week and at present; they also used more often flooded latrines and shared them with someone with diarrhea. Non-cholera diarrhea cases were more likely to receive the OCV than cholera-confirmed cases. See Table S1.

# Table S1. Characteristics of the cholera patients, non-cholera diarrhea patients and matched controls.

|  | **Cholera cases** | **p-value** | **Non cholera diarrhea cases** | **p value**** | | **Matched controls** |
| --- | --- | --- | --- | --- | --- | --- |
|  | **(n=66)** |  | **(n=145)** |  | **n=330 (%)** | |
| Mean age (standard deviation) | 22.7 (16.2) | 0.545 | 21.1 (19.1) | 0.93 | 22.6 (15.2) | |
| Female sex (%) | 28 (42.4) | 0.151 | 77 (53.1) | 1 | 140 (42.4) | |
| Mean household size (standard deviation) | 5.8 (3.4) | 0.113 | 5.2 (2.4) | 0.89 | 5.9 (2.6) | |
| Educational level and literacy (%) |  |  |  |  |  | |
| Illiterate | 13 (19.7) | 1 | 38 (26.2) | 1 | 52 (15.8) | |
| No formal education | 1 (1.5) | 0.764 | 2 (1.4) | 0.72 | 6 (1.8) | |
| Primary | 21 (31.8) | 0.455 | 45 (31.0) | 0.42 | 115 (34.8) | |
| Secondary | 31 (47.0) | 0.144 | 51(35.2) | 0.58 | 152 (46.1) | |
| University | 0 (0) | 0.985 | 9 (6.2) | 0.98 | 5 (1.5) | |
| Health center as first structure for diarrhea treatment (%) | 64 (97.0) | 0.223 | 144 (99.3) | 0.11 | 328 (99.4) | |
| Household member with cholera in the last week (%) | 20 (30.8) | <0.001 | 9 (6.2) | <0.001 | 9 (2.7) | |
| Household member with cholera now (%) | 13 (20.6) | <0.001 | 5(3.5) | <0.001 | 6 (1.8) | |
| Uses safe drinking water source* (%) | 34 (53.1) | 0.705 | 74 (53.2) | 0.43 | 153 (47.7) | |
| Water treated last week (boiled, chlorinated, filtered (%) |  |  |  |  |  | |
| Never | 32 (49.2) | 1 | 52 (36.6) | 1 | 123 (37.3) | |
| Sometimes | 25 (38.5) | 0.214 | 61 (43.0) | 0.15 | 146 (44.2) | |
| Always | 8 (12.3) | 0.08 | 29 (20.4) | 0. 11 | 61 (18.5) | |
| Shares drinking water source with cholera patient in the last week | 17 (28.8) | <0.001 | 9 (7.4) | <0.001 | 25 (8.3) | |
| Ate street food in past week (%) | 52 (80.0) | 0.333 | 104 (73.8) | 0.66 | 252 (77.5) | |
| Has soap available (self-reported) (%) | 45 (69.2) | 0.513 | 106 (73.6) | 0.72 | 221 (67.0) | |
| Has improved sanitation* (self-reported) (%) | 3 (4.6) | 0.423 | 11 (7.6) | 0.44 | 24 (7.3) | |
| Latrine used is flooded (self-reported) (%) | 8 (14.3) | 0.047 | 7 (5.3) | 0.007 | 14 (4.5) | |
| Shares latrine with someone with diarrhea (%) | 16 (25.4) | 0.006 | 15 (10.6) | <0.001 | 21 (6.5) | |
| * as defined by WHO/UNICEF Joint Monitoring Program | | | | | | |
| **P-value from univariable conditional logistic regression adjusting for matching factors | | | | | | |

Cholera confirmed cases were more likely than controls to have had at least one household member with cholera in the week prior to the interview, to share drinking water with cholera patient in the week prior to the interview, to use a flooded latrine and to share a latrine with someone with diarrhea in the week prior to the interview. See Table S1.

Women were over-represented in members of the cohort. Members of the cohort had less risky behavior regarding the management of their drinking water: they treated it more frequently, used factory bottles or safe drinking water sources. However, they reported sharing water, a household, or latrine with someone with diarrhea less frequently than cases. They also reported a neighbor with diarrhea during the previous week less frequently. See Table S2.

Table S2: Characteristics of cohort members and confirmed cases.

|  | **Cohort** | **Cases** | **p-value** |
| --- | --- | --- | --- |
|  | **(n=909)** | **(n=66)** |  |
| Mean age (sd) | 22.9 (15.9) | 22.7 (16.2) | 0.916 |
| Female sex (%) | 557 (61.2) | 28 (42.4) | 0.003 |
| Education (%) |  |  |  |
| Illiterate | 94 (10.5) | 13 (19.7) | 0.094 |
| No formal education | 12 (1.3) | 1 (1.5) | 0.094 |
| Formal education | 789 (88.2) | 52 (78.8) | 0.094 |
| Water treatment type (%) |  |  |  |
| Chlorine | 216 (63.2) | 24 (85.7) | 0.022 |
| Boiling | 109 (31.9) | 4 (14.3) | 0.022 |
| Filter | 17 (4.9) | 0 (0) | 0.022 |
| Drinking water from a pump, | 496 (80.4) | 16 (45.7) | <0.001 |
| a factory filled bottle, or tap (%) |  |  |  |
| Water treatment (%) |  |  |  |
| Never | 284 (31.4) | 32 (49.2) | <0.001 |
| Sometimes | 300 (33.3) | 25 (38.5) | <0.001 |
| Always | 318 (35.3) | 8 (12.3) | <0.001 |
| Use safe drinking water source* (%) | 496 (80.4) | 16 (45.7) | <0.001 |
| Neighbor suffering from | 61 (6.2) | 13 (25.9) | <0.001 |
| diarrhea the last week (%) |  |  |  |
| Shares water with someone with diarrhea(%) | 6 (0.7) | 17 (28.8) | <0.001 |
|  |  |  |  |
| Shares latrine with someone with diarrhea(%) | 18 (2.1) | 16 (25.3) | <0.001 |
| Cholera in household last week(%) | 3 (0.3) | 23 (37.3) | <0.001 |
| Shares water or latrine with someone with diarrhea or had cholera in the household last week (%) | 25 (3.8) | 27 (46.5) | <0.001 |
| Defecate in toilet inside the house (%) | 161 (17.7) | 3 (4.5) | 0.012 |
| Number of children under 5 (%) | 1.7 (0.8) | 2.06 (0.95) | 0.001 |
| Number of children under 5 | 1.6 (0.7) | 2 (0.93) | <0.001 |
| vaccinated against measles (%) |  |  |  |

* defined by WHO/UNICEF Joint Monitoring Program

# Model selection for the multivariate analysis: Adjustment for confounder and effect modifier.

For the TNCC, during the bivariable analysis process with well-known risk factors for cholera infection, the variables associated (p<0.2) with the outcome were: “sex”, “household size”, “education”, “have had at least one household member with cholera in the week prior to the interview”, “have at least one household member with cholera at the time of the interview”, “frequency of water treatment”, “latrine used is flooded” and “share the latrine with someone with diarrhea” (See table S1). Variables associated with the exposure were: “age”, “education”, “have had at least one household member with cholera in the week prior to the interview”, “frequency of water treatment” and “has soap available” (See table S3).

**S3 Characteristics of vaccine recipients and those who did not receive the vaccine by TNCC and MCC design.**

|  | **Test Negative Case Control** | | | **Matched Case Control** | | |
| --- | --- | --- | --- | --- | --- | --- |
|  | **non vaccinated** | **vaccinated** | **p-value** | **non vaccinated** | **vaccinated** | **p-value** |
| **N** | 169 | 42 |  | 349 | 47 |  |
| **mean age (sd)** | 24.35 (18.40) | 10.55 (12.58) | <0.001 | 22.5 (1558.97) | 23.26 (1330.16) | 0.749 |
| **female sex** | 81 (47.93) | 24 (57.14) | 0.287 | 146 (41.83) | 22 (46.81) | 0.518 |
| **mean household size (sd)** | 5.38 (287.41) | 5.36 (241.76) | 0.967 | 5.88 (273.36) | 5.85 (316.9) | 0.948 |
| **illiterate** | 30 (17.75) | 21 (50) | 1 | 62 (17.77) | 3 (6.38) | 1 |
| **no formal education** | 3 (1.78) | 0 (0) | 0.986 | 6 (1.72) | 1 (2.13) | 0.315 |
| **primary** | 54 (31.95) | 12 (28.57) | 0.007 | 120 (34.38) | 16 (34.04) | 0.118 |
| **secondary** | 74 (43.79) | 8 (19.05) | <0.001 | 156 (44.7) | 27 (57.45) | 0.042 |
| **university** | 8 (4.73) | 1 (2.38) | 0.117 | 5 (1.43) | 0 (0) | 0.99 |
| **health centre for diarrhea** | 166 (98.22) | 42 (100) | 0.987 | 345 (98.85) | 47 (100) | 0.985 |
| **cholera in household last week** | 26 (15.57) | 3 (7.14) | 0.186 | 25 (7.23) | 4 (8.51) | 0.773 |
| **cholera in household last now** | 15 (9.2) | 3 (7.32) | 0.704 | 16 (4.62) | 3 (6.38) | 0.6 |
| **uses safe drinking water source** | 87 (54.04) | 21 (50) | 0.724 | 169 (50) | 18 (38.3) | 0.202 |
| **water treatment (never)** | 74 (44.85) | 10 (23.81) | 1 | 136 (39.08) | 19 (40.43) | 1 |
| **water treatment (sometime)** | 67 (40.61) | 19 (45.24) | 0.082 | 150 (43.1) | 21 (44.68) | 0.995 |
| **water treatment (always)** | 24 (14.55) | 13 (30.95) | 0.004 | 62 (17.82) | 7 (14.89) | 0.649 |
| **share water cholera** | 22 (15.6) | 4 (10.26) | 0.404 | 40 (12.62) | 2 (4.76) | 0.154 |
| **ate street food** | 122 (74.39) | 34 (80.95) | 0.378 | 267 (77.62) | 37 (80.43) | 0.665 |
| **has soap available** | 127 (76.05) | 24 (57.14) | 0.016 | 233 (66.95) | 33 (70.21) | 0.655 |
| **has improved sanitation** | 13 (7.78) | 1 (2.38) | 0.238 | 25 (7.18) | 2 (4.26) | 0.461 |
| **Latrine flooded** | 11 (7.33) | 4 (10.81) | 0.488 | 22 (6.67) | 0 (0) | 0.985 |
| **shares latrine with  someone w/ diarrhea** | 26 (16.05) | 5 (11.9) | 0.384 | 33 (9.62) | 4 (8.89) | 0.885 |

**“Education”**, “**frequency of water treatment”** and “**have had at least one household member with cholera in the week prior to the interview”** variables had p-values lower than 0.2 when compared with both the outcome (cholera) and with the vaccination status.

The two potential confounders identified (“share the drinking water source with a cholera patient” and “household member with cholera in the previous week”) were likely measuring a similar dimension of the risk of getting cholera infection, which is reflected in a highly significant fisher-exact test when exploring the association between this two variables (p<0.001). We decided to combine both variables in a single combined variable called “**contact”** that considers as exposed those who had a household member with cholera in the previous week or those who shared the drinking water source with a cholera patient. We also included in the multivariate model the variable “age” since can be a confounder associated with both the vaccine coverage and the VE.

For the **MCC analysis**, during the bivariable analysis process the variables associated (p<0.2) with cholera infection were: “seek a health center for diarrhea treatment”, “have had at least one household member with cholera in the week prior to the interview”, “have at least one household member with cholera at the time of the interview”, “frequency of water treatment”, “share the drinking water source with a cholera patient”, “latrine used is flooded” and “share latrine with someone with diarrhea in the week prior to the interview” (See table S1). Variables associated with the exposure were: “education”, “use safe drinking water source” and “share the drinking water source with a cholera patient”. (See table S2)

The only variable showed p-values lower than 0.2 when compared with both the outcome (cholera) and with the vaccination status was **“share the drinking water source with a cholera patient”.** From the model developed for each variable, **“household member with cholera in the previous week”** variable modified the crude vaccine effectiveness from 85% to 92%.

The final adjusted model includes this combined variable as a possible confounder. In addition, considering that the cases in main analysis could come from both targeted and not targeted areas for vaccination, which were likely at different baseline for cholera spread, we decided to include an strata in the conditional logistic regression model that considers whether people where residing or not in areas targeted by the mass vaccination campaign.

**S4.** **Characteristics of vaccine recipients and those who did not receive the vaccine for the CC design.**

|  | **Not vaccinated with OCV** | **Vaccinated with OCV** | **p-value** | **Relative** |
| --- | --- | --- | --- | --- |
|  | **(n=614)** | **(n=361)** |  | **VE variation (%)** |
| Mean age (sd) | 24.5 (15.3) | 20.2 (16.6) | 0 | -1.10% |
| Female sex (%) | 56.5 | 65.9 | 0.004 | 5.80% |
| Education (%) |  |  |  |  |
| Illiterate | 8.5 | 16.3 | 0.001 | -7.60% |
| No formal education | 1.1 | 1.7 | 0.001 | -7.60% |
| Formal education | 90.3 | 82 | 0.001 | -7.60% |
| Water treatment type (%) |  |  |  |  |
| Chlorine | 59.7 | 74.2 | 0.001 | -16.40% |
| Boiling | 33.6 | 25 | 0.001 | -16.40% |
| Filter | 6.7 | 0.8 | 0.001 | -16.40% |
| Drinking water from a pump, | 77.6 | 78.1 | 0.88 | 0.00% |
| a factory filled bottle, or tap (%) |  |  |  |  |
| Water treatment (%) |  |  |  |  |
| Never | 33.8 | 30.8 | 0.432 | 2.00% |
| Sometimes | 32.9 | 34.7 | 0.432 | 2.00% |
| Always | 33.3 | 34.5 | 0.432 | 2.00% |
| Use safe drinking water source* (%) | 77.6 | 78.1 | 0.88 | 0.00% |
| Neighbor suffering from | 8.6 | 5.7 | 0.122 | 7.70% |
| diarrhea the last week (%) |  |  |  |  |
| Shares water with someone | 4.3 | 1.5 | 0.052 | 16.60% |
| with diarrhea(%) |  |  |  |  |
| Shares latrine with someone | 5.3 | 2 | 0.041 | 19.10% |
| with diarrhea(%) |  |  |  |  |
| Cholera in household | 3.5 | 1.7 | 0.104 | 3.20% |
| last week(%) |  |  |  |  |
| Shares water or latrine with | 8.4 | 3.8 | 0.021 | 37.10% |
| someone with diarrhea or had |  |  |  |  |
| cholera in the household last week (%) |  |  |  |  |
| Defecate in toilet inside the house (%) | 23.5 | 5.5 | 0 | -14.70% |
| Number of children under 5 (%) | 1.7 (0.81) | 1.8 (0.8) | 0.173 | -2.10% |
| Number of children under 5 vaccinated against measles (%) | 1.6 (0.78) | 1.7 (0.7) | 0.052 | -6.80% |

* defined by WHO/UNICEF Joint Monitoring Program

We initially considered for inclusion in the multivariable analysis the variables with a p-value below 0.2 in univariate analysis or that differed significantly between cases and participants to the cohort. Secondarily we assessed the impact of their inclusion in a simple model where only vaccination status was forced on the unadjusted VE.

Sex, in addition to vaccination status, was forced in every multivariable model considered because of the significant over-representation of women in the cohort. After careful consideration, we decided to exclude from the multivariable analysis the variables assessing direct or indirect contacts with someone with diarrhea such as sharing latrine, water, or a household or attending to his/her funeral. A substantial part of the cohort participants were recruited after the bulk of the cases were included, at a period where the outbreak incidence was sharply decreasing. Therefore. their risk of being in contact with someone with diarrhea was likely much lower than for cases. We then excluded those variables in the multivariable analysis to avoid artificially overestimating their RR and biasing our VE estimates.

The various variables referring to water management, that differed between cases and the cohort, were correlated among themselves so we only considered the more global one using a clear international definition : access to safe water as defined by WHO/UNICEF Joint Monitoring Program. A similar logic was applied to the variables assessing latrine/defecation management.

Eventually, AIC and BIC agreed and privileged a final model without variables assessing the level of education.

**Table S5. Summary of the variables included in the multivariate models**

|  | **TNCC** | **MCC** | **CC** |
| --- | --- | --- | --- |
| **Crude Vaccine Effectiveness (95% Cl)** | 87.1% (62.4-97.0) | 84.7% (27.0-96.6) | 86.7% (56.6-95.9) |
| **Variables included as possible confounders** |  |  |  |
| Age | x |  | x |
| Sex |  |  | x |
| Education level | x |  |  |
| Frequency of treating the drinking water | x |  |  |
| Household member with cholera in the previous week | x | x |  |
| Shared the drinking water source with a cholera patient | x | x |  |
| Nº of children under 5 years living in the household |  |  | x |
| Access to safe water |  |  | x |
| Place of defecation |  |  | x |
| **Adjusted Vaccine Effectiveness (95% CI)** | 80.2% (16.9-95.3) | 88.9% (42.7-97.8) | 89.4% (64.6-96.9) |

# Missing values

Databases, for both case control study and cohort, did not contain any missing data for the outcome or the vaccination status. Table S6 shows variables with missing data that were tested as possible confounders.

Table S6. Percentage of missing among variables tested as possible confounders.

|  | **Controls** | **Cholera Cases** | **Suspected Cholera Cases** | **Cohort** |
| --- | --- | --- | --- | --- |
|  | **(n=330)** | **(n=66)** | **(n=211)** | **(n=906)** |
| Household size | 0.0% | 1.5% | 0.9% | 0.1% |
| Educational level | 0.0% | 0.0% | 0.0% | 1.5% |
| Seeking care at health center for diarrhea | 0.0% | - | - | 0.0% |
| Household member with cholera in the last week | 0.6% | 1.5% | 0.9% | 0.5% |
| Household member with cholera now | 0.0% | 4.5% | 3.3% | 0.1% |
| Use safe drinking water source* | 1.8% | 1.5% | 4.3% | 24.4% |
| Water treatment | 0.0% | 1.5% | 1.9% | 0.8% |
| Shares drinking water source with cholera patient | 9.1% | 10.6% | 14.7% | 7.4% |
| Ate street food in the past week | 1.5% | 1.5% | 2.4% | 0.3% |
| Had soap available (self-reported) | 0.0% | 1.5% | 0.9% | 0.0% |
| Had improved sanitation* (self-reported) | 0.0% | 1.5% | 0.9% | 0.0% |
| Latrine used is flooded (self-reported) | 5.2% | 15.2% | 11.4% | 0.0% |
| Shares latrine with someone with diarrhea | 1.5% | 4.5% | 3.3% | 5.6% |
| * as defined by WHO/UNICEF Joint Monitoring Program | | | | |

The crude analysis was not affected by the missing data since the database did not contain any missing information for the outcome, the exposure or the matched set. For the multivariate analysis, the two variables identified as possible confounders contained missing data and we analyzed the possible influence of the missing data in the adjusted vaccine effectiveness estimates. We explored three different strategies to handle the missing values in the multivariate analysis: (i) removing the entries with missing values for any of the variables of interest, (ii) considering the missing data as a dummy variable, (iii) imputing the missing data using multiple imputation with chained equations (MICE)(27,28).

Following this process, we created 100 imputed data sets that were used for the multivariate analyses. The table S7 shows the adjusted vaccine effectiveness estimates for each strategy. We include in the main manuscript as the adjusted vaccine effectiveness the point estimate and the confidences intervals obtained with missing data imputed using multiple imputation with chained equations as the adjusted vaccine effectiveness.

**Table S7. Adjusted vaccine effectiveness estimates considering different strategies to handle the missing data by design**.

|  | **TNCC** | | **MCC** | | **CC** | |
| --- | --- | --- | --- | --- | --- | --- |
|  | **Adjusted VE*** | **95% CI** | **Adjusted VE**** | **95% CI** | **Adjusted VE†** | **95% CI** |
| Entries with missing data excluded | 81.2% | (19.2-95.6) | 86.5% | (29.1-97.4) | 94.0% | (52.5-99.2) |
| Missing values as a dummy variable | 80.9% | (20.5-95.4) | 87.4% | (30.9-97.7) | 89.2% | (64.4-96.7) |
| Multiple imputation of missing values | 80.2% | (16.9-95.3) | 88.9% | (42.7-97.8) | 89.4% | (64.6-96.9) |

***TND**: Vaccine effectiveness adjusted by age, education level, frequency of treating the drinking water and contact (combined variable that considers those who had a household member with cholera in the previous week or shared the drinking water source with a cholera patient as ‘exposed‘). Living in a vaccinated area was included as a stratification variable in the conditional logistic regression model.

****MCC** Adjusted by contact. Living in a vaccinated area was included as a stratification variable in the regression model.

†**CC**: Vaccine effectiveness was adjusted by age, sex, number of children under 5 years of age living in the household, access to safe water, and the place of defecation.

# Sensitive analysis considering the distance between the control and their case

We used spatial matching in the MCC design to help control for local vaccine coverage and infection risk, which is in theory, should lead to estimates of the direct vaccine effectiveness. However, it isn’t clear what the most appropriate spatial scale for matching might be . To assess the sensitivity of our results to the distance between the control and their case (i.e., the spatial scale), we estimated the vaccine effectiveness in the vaccinates areas using two alternative vaccination classification schemes; (1) excluding those pairs at a distance more than 150m (2) excluding those pairs at a distance more than 300m. With both of these approaches, we found that our estimates of VE were within 4% of the original estimates.

**Table S8. Sensitivity analysis on the vaccination status in participants living in a vaccinated area excluding the pairs control with a distance between the control and their case of more than 150 m, more than 300 and without any distance exclusion**
